# Posterior reversible encephalopathy syndrome

**DOI:** 10.1007/s00415-016-8377-8

**Published:** 2017-01-04

**Authors:** Marlene Fischer, Erich Schmutzhard

**Affiliations:** 1 Department of Anesthesiology, University Medical Center Hamburg-Eppendorf, Martinistrasse 52, 20246 Hamburg, Germany; 2 Department of Neurology, Medical University of Innsbruck, Innsbruck, Austria

**Keywords:** Encephalopathy, Vasogenic edema, Hypertensive encephalopathy, Chemotherapy, Preeclampsia

## Abstract

The posterior reversible encephalopathy syndrome (PRES) is a neurological disorder of (sub)acute onset characterized by varied neurological symptoms, which may include headache, impaired visual acuity or visual field deficits, disorders of consciousness, confusion, seizures, and focal neurological deficits. In a majority of patients the clinical presentation includes elevated arterial blood pressure up to hypertensive emergencies. Neuroimaging, in particular magnetic resonance imaging, frequently shows a distinctive parieto-occipital pattern with a symmetric distribution of changes reflecting vasogenic edema. PRES frequently develops in the context of cytotoxic medication, (pre)eclampsia, sepsis, renal disease or autoimmune disorders. The treatment is symptomatic and is determined by the underlying condition. The overall prognosis is favorable, since clinical symptoms as well as imaging lesions are reversible in most patients. However, neurological sequelae including long-term epilepsy may persist in individual cases.

## Introduction

Posterior reversible encephalopathy syndrome (PRES) is a neurological disorder characterized by a range of neurological signs and symptoms and distinctive neuroimaging findings reflecting vasogenic edema [1]. Both clinical and imaging characteristics are usually reversible [2]. On average, about 40% of all patients diagnosed with PRES require intensive care monitoring and treatment due to severe complications such as status epilepticus, cerebral ischemia, intracerebral hemorrhage or intracranial hypertension [3].

The syndrome was first described in 1996 by Hinchey and colleagues who reported on a series of 15 patients with neurological signs and symptoms including headache, seizures, visual disturbance and other focal neurological deficits [4]. Moreover, computed tomographic (CT) or magnetic resonance imaging (MRI) alterations suggestive of cerebral edema were observed predominantly in the posterior regions [4]. Since this first description of PRES numerous case reports and case series, as well as retrospective observational studies describing the syndrome have been published. Importantly, no randomized controlled studies have been performed, a fact that has to be taken into account when discussing epidemiological data, diagnostic criteria and treatment recommendations.

Epidemiological data in particular should be interpreted with caution, since the syndrome may still be significantly underdiagnosed as the condition can be hard to confirm. PRES has been reported in almost all age groups, from children to older adults, but most frequently in young- or middle-aged adults with a preponderance of female patients, which might be attributable to etiological aspects [5, 6].

## Etiology and pathophysiological considerations

**Fig. 1:**
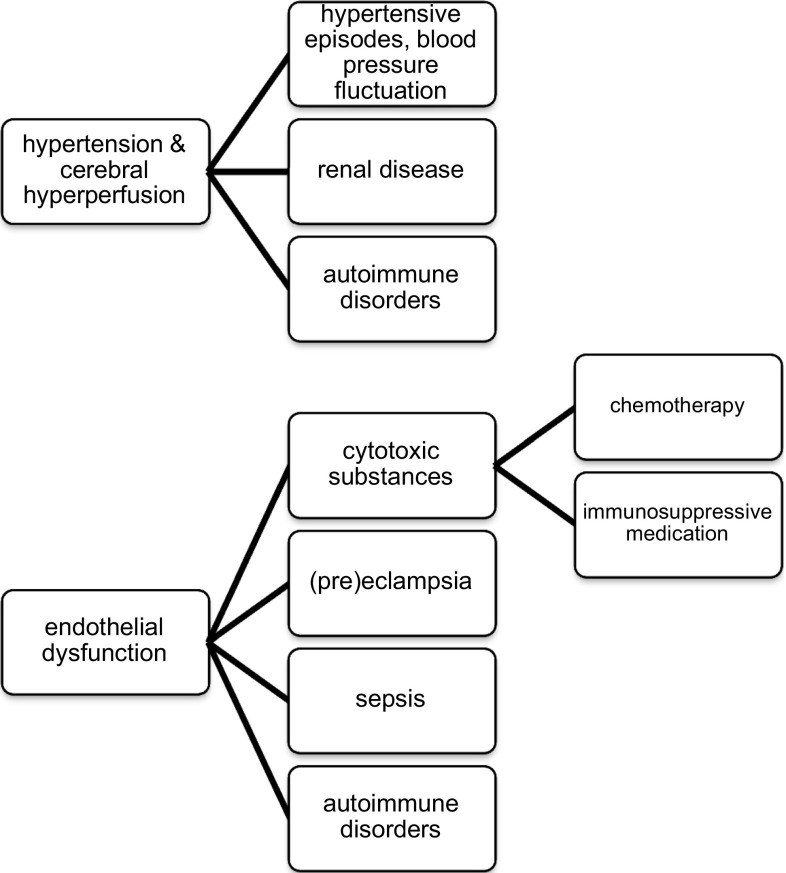
The two main hypotheses explaining the pathophysiology of posterior reversible encephalopathy and associated conditions

There are two leading theories regarding the pathophysiology of PRES (Fig. 1) [7]. The first hypothesis proposes a rapid increase of arterial blood pressure up to a hypertensive crisis or emergency, which has been observed in a majority of patients at PRES onset [1]. According to this hypothesis, elevation of blood pressure levels above the upper autoregulatory limit leads to cerebral hyperperfusion, which may cause vascular leakage and vasogenic edema [8]. Increased cerebral perfusion pressure contributes to additional blood–brain barrier dysfunction causing extravasation of plasma and macromolecules through tight-junction proteins [7].

Cerebrovascular autoregulation is supposed to preserve a continuous cerebral blood flow independently of systemic blood pressure fluctuations [9]. This is ensured by vasodilation of the cerebral arteries during hypotensive episodes. In contrast, during periods of hypertension, this results in cerebral vasoconstriction. This adaptive mechanism is mainly regulated by pressure and carbon dioxide reactivity, as well as the release of vasoactive substances such as nitric oxide, thromboxane A_2_ or endothelin-1 from the vascular endothelium [1].

In healthy individuals a continuous cerebral blood flow can be maintained between the lower and upper autoregulatory limits, usually a cerebral perfusion pressure between 50 and 150 mmHg [10]. Various conditions such as arterial hypertension, acute fluctuations of blood pressure or autonomic activity may induce changes of these autoregulatory thresholds. This may lead to increased vulnerability of the cerebral circulation and predispose to cerebral ischemia during periods of hypotension on the one hand, or cerebral hyperperfusion and vascular leakage on the other, when blood pressure rises above the upper autoregulatory limit [11, 12]. The “hyperperfusion theory” is supported by observations of elevated or fluctuating blood pressure, or hypertensive episodes in a majority of patients with PRES at disease onset [3].

The posterior areas of the cerebral hemispheres seem to be particularly susceptible, which is supported by clinical as well as imaging findings. This might be caused by a reduced density of sympathetic innervation in the posterior, compared to the anterior, circulation, the latter being more densely innervated by the superior cervical ganglion [1]. This may prevent excessive vasodilation, which could reduce the risk of cerebral hyperperfusion in these areas compared to the posterior regions.

However, arguing against this hypothesis is that about 30% of patients with PRES show normal or only slightly elevated blood pressure values that do not necessarily exceed the normal upper autoregulatory limit, as would be expected in the context of cerebral hyperperfusion [13]. Thus, the theory of hypertensive episodes and cerebral hyperperfusion as the underlying pathological condition in PRES is still a matter of controversy.

The second theory regarding the cause of PRES is that the syndrome is triggered by endothelial dysfunction caused by circulating endogenous or exogenous toxins [7]. Arguing for this hypothesis, PRES is frequently observed in patients with (pre)eclampsia, sepsis or during treatment regimens with immunosuppressive agents or cytotoxic medication [14–16]. The common factor in these diverse conditions is the presence of endogenic (preeclampsia, sepsis) or exogenic (chemotherapy, immunosuppressive agents) toxins causing endothelial dysfunction [17]. One of the key features of the vascular endothelium is the preservation of vascular integrity by inter-endothelial adhesion molecules. Circulating toxins could trigger vascular leakage and edema formation, and additionally lead to endothelial activation resulting in the release of immunogenic and vasoactive substances [17]. Vasoconstrictive agents released by vascular endothelial cells are thought to mediate cerebral vasospasm, which is frequently observed in PRES patients [2]. In this “toxic” theory, blood pressure elevations occur as a consequence of primary endothelial dysfunction. A variation on the “toxic/immunogenic” theory is that the trigger is the excessive release of pro-inflammatory cytokines resulting in endothelial activation, release of vasoactive agents, increased vascular permeability and edema formation. This mechanism is regarded as the key feature causing PRES in patients with autoimmune disorders or sepsis [17].

Apart from arterial hypertension, a variety of conditions have been linked to the diagnosis of PRES. Etiologies may be manifold; however, a clear correlation between clinical signs and symptoms, lesion site or specific trigger factors has not been observed [2, 5]. PRES has been frequently reported in patients receiving immunosuppressive medication after solid organ, bone marrow or stem cell transplantation [18, 19]. The incidence of PRES after solid organ transplantation is reported to be between 0,4 and 6%, whereas up to 8% of patients after bone marrow transplantation may be affected [18, 20].

Compared to solid organ transplantation immunosuppressive medication is usually administered at a higher dose with bone marrow or stem cell transplantation, possibly explaining the higher incidence of PRES after non-solid organ transplantation. However, it is unclear whether PRES is linked to the dose of causative agents. Plasma levels of immunosuppressive substances do not necessarily correlate with the severity of clinical signs or imaging findings [20, 21]. Moreover, PRES has been observed up to several months after administration of cytotoxic agents [20]. Adding to this controversy, there are numerous reports of PRES in patients with plasma concentrations of immunosuppressants within the therapeutic range. Nevertheless, tapering off or reducing the dosage of causative agents usually leads to clinical improvement and/or a reduction in lesion size [20]. This observation supports a positive correlation between the dose of the offending agent and the neurological/radiological manifestations.

The exact mechanism of how specific substances may cause this form of encephalopathy is unknown. Numerous authors have reported calcineurin inhibitors to be linked with PRES development [22–24]. These substances are well-known for their neurotoxic properties, which have been attributed to the release of vasoconstrictive substances, aggravation of hypomagnesemia, and arterial hypertension [25, 26]. In a retrospective study, Hammerstrom and colleagues observed an average increase of 35% in the mean arterial blood pressure under a Tacrolimus regimen [21]. Adding to the reported effects, polymorphisms in the multidrug resistance protein 1 gene may allow central nervous system dissemination of these substances [27]. Importantly, Tacrolimus but also antiangiogenic drugs such as Bevacizumab, Sunitinib or Sorafenib may mediate increased vascular permeability, thereby contributing to edema formation [1].

Autoimmune disorders have been frequently reported in the context of PRES. Fugate and colleagues report a history of autoimmune disease in 45% of patients in a retrospective study of 120 cases [5]. Several explanations have been provided for this linkage [5, 17]. As is the case in post-transplant patients, immunosuppressive medication may play an important role. Additionally, (auto)immunologic reactions may trigger endothelial activation by excessive cytokine release followed by vascular leakage of proteins and fluid into the interstitial space.

Renal disease and preeclampsia have also been linked to PRES. Impaired renal function has been reported in 55% of all patients with PRES [1]. However, it is unclear whether accompanying arterial hypertension or renal dysfunction itself is the primary causal factor.

PRES occurs frequently in the setting of preeclampsia or eclampsia [28]. In a retrospective study, PRES was found in more than 90% of eclamptic and about 20% of preeclamptic patients with neurological symptoms [16]. Compared with pregnant women with eclampsia or preeclampsia without PRES, significant elevations of hematocrit, serum creatinine, aspartate transaminase, alanine transaminase and lactate dehydrogenase values were noted [16].

## Clinical findings

PRES is characterized by a variety of neurological symptoms, usually going along with elevated arterial blood pressure. The onset may be acute or subacute, with symptoms developing within a few hours up to several days or even weeks [1].

Patients may present with signs of encephalopathy, including quantitative and qualitative disorders of consciousness such as cognitive deficits or stupor, somnolence or coma [2]. Epileptic seizures, focal as well as generalized, are very common, and have been observed in about two third of all patients [3, 29]. In 3–13% of cases seizures may result in status epilepticus, which is one of the most severe and potentially life threatening complications of PRES [3, 30].

**Fig. 2:**
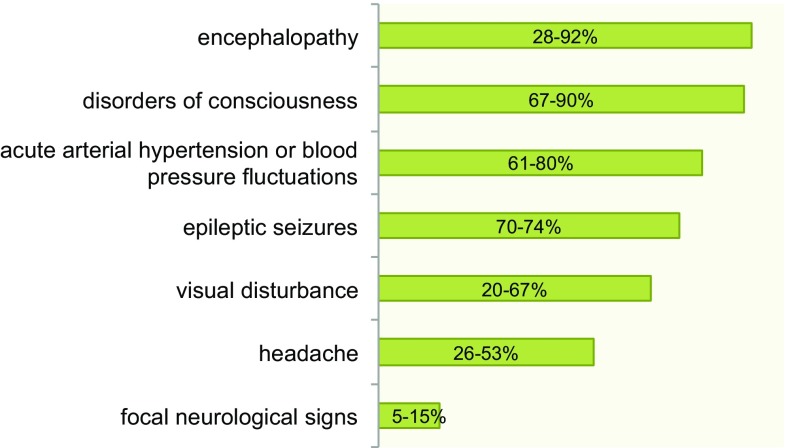
Incidence of neurological signs in patients with posterior reversible encephalopathy syndrome

In accordance with the frequent involvement of the occipital lobes, visual disturbances such as a deterioration of visual acuity, visual field deficits including hemianopia and cortical blindness or visual hallucinations can be observed in about two third of all PRES patients [4]. Less specific neurological symptoms include headache, nausea, vomiting and disorders of consciousness. Depending on the location of the lesions, focal neurological deficits have been reported in 5–15% [1, 31, 32]. Some case reports have described myelopathic symptoms in patients with spinal cord involvement [33]. An overview of the most common clinical findings is provided in Fig. 2.

## Diagnosis

**Fig. 3:**
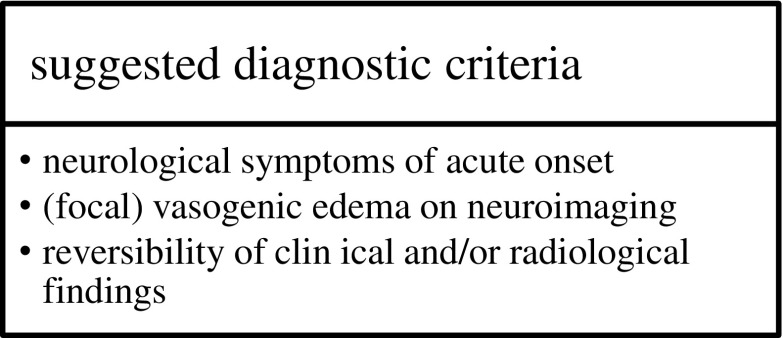
Suggested criteria for the diagnosis of posterior reversible encephalopathy syndrome Modified after Fugate et al. (2010) [5]

Established diagnostic criteria have been lacking so far and clinical as well as imaging findings are often not specific (Table 1). Therefore, the diagnosis of PRES can often only be made after excluding important other diagnoses. The presence of neurological symptoms of acute onset, concurrent blood pressure fluctuations, vasogenic edema as the leading neuroimaging finding and a clinical context of associated comorbidities or trigger factors are suggestive of PRES. Fugate et al. suggested the following criteria for the diagnosis of PRES: neurological symptoms of acute onset, neuroimaging abnormalities of (focal) vasogenic edema and the reversibility of clinical and/or radiological findings (see Fig. 3) [5].

**Table 1:** Diagnostic findings in patients with posterior reversible encephalopathy syndrome

Diagnostic tool	Finding
Laboratory data	Hypomagnesemia
	Lactate dehydrogenase ↑
	Liver function parameters ↑
	Creatinine ↑
	Albumin ↓
Cerebrospinal fluid	Albumin ↑
	Albuminocytologic dissociation
EEG	Diffuse theta slowing
	Delta slowing
	Rhythmic delta activity
	Sharp-slow wave activity
	Periodic lateralizing epileptiform discharges
	Diffuse or focal (symmetric) slowing of background activities
CT and MRI	Vasogenic edema
	Watershed distribution
	Parieto-occipital pattern
	Frontal and temporal lobe involvement
	Subcortical white matter lesions
	Bilateral, frequently symmetric distribution
	Hyperintense T2-weighted and FLAIR sequences
	Iso-, hypo-, or hyperintense lesions on DWI
	Facultative contrast enhancement
	Microbleeds, intracerebral hemorrhage possible
	Increased or decreased ADC values depending/indicating (ir)reversibility of lesions
Angiography	Vasoconstriction, vasospasm (diffuse or focal)

*EEG* electroencephalogram, *CT* computed tomography, *MRI* magnetic resonance imaging, *FLAIR* fluid-attenuated inversion recovery, *DWI* diffusion-weighted imaging, *ADC* apparent diffusion coefficient

Neuroimaging, in particular MRI, is the most important diagnostic tool. Therefore, characteristic neuroimaging findings are discussed in more detail below. Electroencephalography (EEG) may be necessary for the detection of (non convulsive) epileptic seizures, status epilepticus and may also help in the evaluation of encephalopathy [29]. Lumbar puncture is of major importance to exclude encephalitis or leptomeningeal spread in patients with hemato-oncological disease. However, pathological alterations in cerebrospinal fluid (CSF) that are specific for PRES have not been observed. Elevated CSF levels of albumin and an elevated CSF/serum albumin quotient as a manifestation of blood–brain barrier disruption have been reported in a series of 87 patients, whereas pleocytosis was rare [34]. This is in line with a retrospective review of 73 patients with PRES undergoing lumbar puncture [35]. Mild albuminocytologic dissociation was found in all patients with median protein levels of 58 mg/dl [35].

Serum findings are usually not specific. Hypomagnesemia during the first 48 h after onset was reported in a cohort of patients with PRES of varying etiology [36]. Both Gao and Pirker et al. observed decreased serum albumin in up to 85% of patients with PRES of miscellaneous etiology [37, 38].

**Fig. 4:**
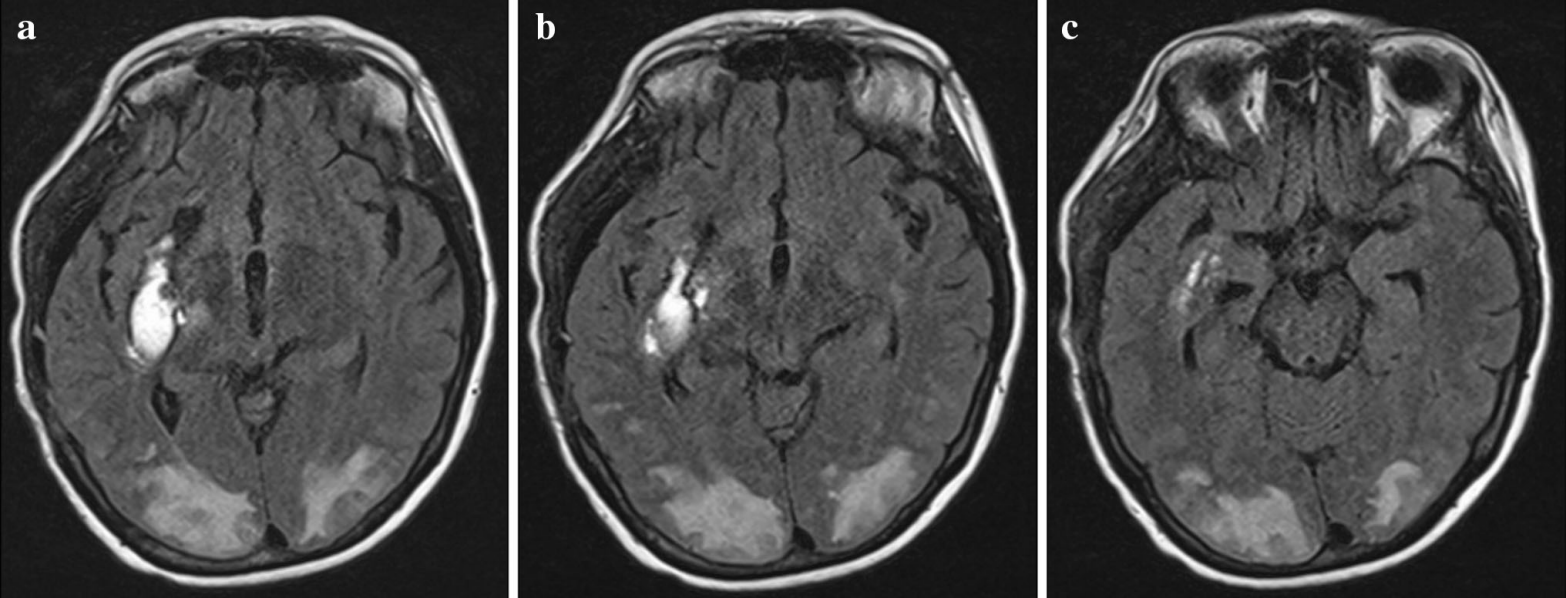
**a**–**c** Axial MR image (fluid-attenuated inversion recovery sequence) demonstrates extensive vasogenic edema in the occipital region bilaterally and right insular hemorrhage

CT scans usually show vasogenic edema with a bihemispheric distribution [2]. MRI is more sensitive displaying hyperintense lesions in T2-weighted or fluid-attenuated inversion recovery (FLAIR) sequences [2]. MRI lesions reflecting vasogenic edema frequently follow a parieto-occipital pattern [15]. Though usually bihemispheric, lesions may be distributed asymmetrically (Fig. 4). Due to the lower density of the white matter, subcortical areas are affected predominantly. However, cortical involvement has also been described [2]. While the parieto-occipital distribution occurs in about 70% of all patients, a frontal sulcus or watershed pattern is also frequently seen [39]. Lesions in other areas such as the cerebellum, brain stem, basal ganglia or the spinal cord are less common [32].

Though rare, diffusion abnormalities can be found as small lesions surrounded by edematous zones. The presence of larger areas of restricted diffusion may be indicative of ischemic stroke. Increased apparent diffusion coefficient (ADC) values on diffusion-weighted imaging (DWI) are characteristic and reflect vasogenic edema [15]. ADC imaging can be of prognostic relevance: higher values have been associated with a reversibility of lesions [15]. By contrast, attenuated ADC values indicate cerebral ischemia and a poor neurological outcome [15]. Contrast enhanced lesions have been found in about 20% of all patients. However, there does not seem to be a clear link to clinical severity or functional outcome [40].

In a retrospective observational study, the presence of microbleeds in susceptibility weighted imaging has been reported as an initial presentation and on follow-up scans [41]. The authors found microhemorrhages in 65% of all cases [41]. As with contrast enhancement, there is no clear correlation with clinical symptoms. Therefore, the clinical relevance of microbleeds in PRES has yet to be determined.

Imaging studies on cerebral perfusion in PRES patients have reported conflicting results. Increased perfusion has been observed in edematous zones confirming the hypothesis of cerebral hyperperfusion as a result of a blood pressure that is above the upper limit of cerebral autoregulation [42]. In contrast, single photon emission computed tomography (SPECT) and MR perfusion demonstrate cerebral hypoperfusion in lesion zones in PRES of varying etiology [43, 44].

Different perfusion patterns in PRES may be explained by the variety of etiological aspects, causing a diverse pathophysiological response. In line with the main hypotheses for PRES pathophysiology, conflicting perfusion patterns may be a result of primary hypertension and cerebral perfusion on the one hand or endothelial dysfunction, cerebral vasoconstriction and/or vasospasm followed by cerebral hypoperfusion on the other hand. Vasculopathy has been observed in MR or conventional angiography. Vasculopathic findings are usually reversible and include cerebral vasoconstriction, vasospasm (both diffuse or focal) and string-of-beads appearances which are usually located in the posterior circulation [2].

One of the most important differential diagnoses of PRES is reversible cerebral vasoconstriction syndrome (RCVS). Both conditions have similar clinical and angiographic findings. As with PRES, RCVS is frequently diagnosed postpartum or after administration of vasoactive substances and vasculopathic alterations may follow a similar distribution pattern [45, 46]. Interestingly, PRES-like lesions have been observed in patients with RCVS, suggesting that both conditions may reflect different manifestations of the same pathology.

## Treatment

The treatment of PRES is symptomatic, since no specific therapeutic strategy is currently available. The management of the underlying disease or pathology leading to PRES development is of major importance.

The management of hypertensive episodes and maintenance of normal blood pressure is an essential component of PRES treatment [1, 15, 47]. However, there is no evidence, based on prospective controlled studies, that strict blood pressure control limits neurologic injury, or results in a regression of clinical or imaging findings. The choice of antihypertensive drugs per se is based on general recommendations for the management of hypertensive crisis or hypertensive emergency [48, 49]. A reduction of blood pressure levels by 25% from baseline values is recommended. As with other conditions, blood pressure fluctuations should be avoided and the continuous administration of antihypertensive drugs under hemodynamic monitoring should be considered [50].

Anticonvulsive treatment is frequently required. There is no general recommendation for the use of specific drugs. Moreover, the optimal duration of antiepileptic drug treatment is unclear. Usually, anticonvulsive medication can be tapered off as soon as the patient is asymptomatic and the imaging lesions have fully reversed [15, 29].

Whenever possible, the elimination of the triggering factor or management of the underlying pathology should be initiated early during the course of the disease [1, 13, 15]. In many cases of PRES, immunosuppressive or cytotoxic medication is identified as the substance responsible for the neurological manifestations. It is still a matter of controversy whether tapering off or immediate discontinuation of the triggering agent is required, or whether reducing the dosage with strict control of serum levels within the therapeutic range is sufficient. Adding to this issue, the most beneficial therapeutic regimen after the neurological symptoms have resolved is unknown. In a retrospective analysis, Hammerstrom et al. compared three interventions after Tacrolimus-induced PRES in pediatric patients following stem cell transplantation [21]. They either: (1) continued Tacrolimus in the same dosage as before the onset of PRES; (2) interrupted Tacrolimus administration for a mean of 12 days; or (3) suspended treatment with Tacrolimus but switched to a different immunosuppressant immediately. Interestingly, there was no difference in mortality between the three groups in this retrospective analysis. Singer et al. continued treatment with the agent in question in 7 out of 17 cancer patients and did not find recurring PRES [51]. In patients with autoimmune disease, further administration of immunosuppressive medication may require a different management than in patients after solid organ or stem cell transplantation. In a review of patients with PRES associated with systemic lupus erythematosus, active disease was found as the initiating trigger and intensification of immunosuppressive therapy was suggested to control neurological manifestations [52].

Due to the fact that magnesium levels are reduced in a high number of patients with PRES, coupled to its known prophylactic anticonvulsive and vasodilating effects, hypomagnesemia should be avoided and serum levels be maintained in the high normal range [15, 36].

In case of cerebral vasospasm or cerebral vasoconstriction, treatment of the vasospasm (either systemically or, if required, through local intra-arterial administration of calcium antagonists) may be initiated at an early stage.

## Prognosis and outcome

The prognosis of PRES is mainly determined by the underlying condition, since the neurological manifestations are reversible in the majority of patients. However, since PRES is often accompanied by severe complications, neurological sequelae may persist.

In a recent retrospective chart review, poor neurological outcome, as defined by a modified Rankin scale score between 2 and 6, was reported in 36% of all patients at hospital discharge [53]. The authors found that preexisting diabetes mellitus and corpus callosum involvement of the PRES-associated lesions were strong predictors of poor outcome. Singer and colleagues observed a complete resolution of neurological signs and symptoms in 84% of cancer patients with PRES [51]. In 81% of cases, neuroimaging findings were reversible on follow-up MRI or CT scans. Mortality rate in their cohort was reported to be 19%. However, no death was directly associated with PRES. In a review of 111 pediatric cases with hematological disease, 19 patients (17%) died of a PRES-associated mortality [54]. Neurological sequelae including epilepsy, motor deficits and mydriasis were observed in another 17 patients. This is in line with a retrospective study in 35 pediatric cases of PRES triggered by cancer treatment, which also reported a long-term requirement for anticonvulsive treatment due to persistent epilepsy in 19% of patients [55]. Persisting epilepsy with seizures occurring one year after PRES onset was reported in two patients out of a cohort of 75 [56]. Heo and colleagues reviewed 102 cases of PRES and found long-term epilepsy in four patients [57]. In contrast, Kastrup et al. described a cohort of 49 patients, 38 of them presenting with seizures during the acute phase [29]. At follow-up none of their patients suffered from persisting epilepsy.

So far factors such as serum markers, CSF or neuroimaging findings in PRES have not been identified as useful in the risk stratification of patients nor as a measure of prognostic relevance. However, in a recent study by Karia et al., MR imaging severity (as defined by McKinney et al. 2007) correlated with clinical outcomes in 135 patients [40].

## Future directions

Although numerous case reports and observational studies have been published since the first description in 1996, many aspects of PRES, in particular on pathophysiology and treatment, remain unclear [4]. Findings on cerebral perfusion in PRES patients are conflicting, since hyperperfusion as well as decreased perfusion have been reported after PRES [42–44]. Future neuroimaging studies should focus on angiographic imaging and perfusion patterns to characterize cerebral hemodynamics during PRES that may vary depending on etiological aspects or disease progress. Further, non-invasive continuous monitoring of the cerebrovascular autoregulation may aid in the optimal hemodynamic management and the definition of individual blood pressure targets maintaining a constant cerebral blood flow within the limits of cerebral autoregulation [58].

Although there is consensus on the elimination of the etiological factor in PRES induced by cytotoxic medication, further management of immunosuppressants or chemotherapy remains a challenging issue that is usually decided on an individual basis. For clarification, future studies should address several questions: (1) Does the medication causing PRES symptoms have to be eliminated persistently? (2) If not, what is the optimal duration for treatment interruption? (3) Are patients at risk for recurring PRES? (4) Is there a linear correlation between clinical symptoms and substance dose?

In conclusion, PRES-associated clinical signs and symptoms and neuroimaging lesions are reversible in the majority of patients. The prognosis is mainly determined by the underlying pathology. However, neurological sequelae, in particular epilepsy, may persist in individual cases and may require long-term treatment. So far, specific prognostic factors have not been identified. The severity of MR imaging lesions including ADC values may be an important parameter determining long-term prognosis.
